# The Concept of Neuromuscular Repatterning in Dancers: A Systematic Review

**DOI:** 10.3390/healthcare12030402

**Published:** 2024-02-04

**Authors:** Sebastián Gómez-Lozano, Ningyi Zhang, Ross Armstrong, Kiko León, Clare Kelly-Lahon, Pedro Sánchez-González, Ignacio Martínez-González-Moro, María Antonia Hurtado-Guapo, Alfonso Vargas-Macías

**Affiliations:** 1 Performing Arts Research Group, Faculty of Sport, San Antonio Catholic University, 30107 Murcia, Spain; nzhang@alu.ucam.edu (N.Z.); psgonzalez@ucam.edu (P.S.-G.); ahurtado@unex.es (M.A.H.-G.); 2 Rehabilitation and Healthy Lives Research Group, Institute of Health, University of Cumbria, Carlisle CA1 2HH, UK; ross.armstrong@cumbria.ac.uk; 3 Optimization of Training and Sports Performance Research Group, Faculty of Sport Science, University of Extremadura, 10005 Cáceres, Spain; fleon@unex.es; 4 Department of Marketing, Tourism and Sport, Atlantic Technological University, F91 YW50 Sligo, Ireland; clare.kelly-lahon@atu.ie; 5 Research Group of Physical Exercise and Human Performance, Mare Nostrum Campus, University of Murcia, 30100 Murcia, Spain; igmartgm@um.es; 6 Telethusa Centre for Flamenco Research, 11004 Cádiz, Spain; vargas@flamencoinvestigacion.es

**Keywords:** somatic training, imagery, developmental movement, dance injuries

## Abstract

Repatterning is a term that can be used in different fields, including genetics, molecular biology, neurology, psychology, or rehabilitation. Our aim is to identify the key concept of neuromuscular repatterning in somatic training programmes for dancers. A systematic search of eight databases was conducted using the PRISMA (Preferred Reporting Items for Systematic Review and Meta-Analysis) guidelines. The Quality Assessment Tool for Quantitative Studies and the Oxford Levels of Evidence scales were used. The search yielded 1218 results, of which 5 met the inclusion criteria. Five studies (n = 5) were related to psychosomatic health (n = 5), two studies highlighted integration and inter-articular connectivity in movement (n = 2), four studies investigated the neurological component of alignment and efficiency in dance practice (n = 4), and two studies investigated self-confidence (n = 2). Five studies (n = 5) used imagery based on the anatomical and physiological experience of body systems as the main analytical method. Four studies (n = 4) used developmental movement through Bartenieff fundamentals as the main technique for this methodology. Developmental movement and imagery are two methodologies strongly connected to the concept of neuromuscular repatterning in somatic training programmes for dancers. The acquisition of further quantitative experimental or quasi-experimental studies is warranted to better define the level of improvement or impact of neuromuscular repatterning in dancers.

## 1. Introduction

Repatterning is an expression used in different fields of intervention to refer to changes in or reorganisations or adjustments of specific patterns, structures, or processes. The fields of application can be diverse: genetics and molecular biology [1,2,3,4,5,6,7,8,9]; regeneration in cerebellar development [10], brain patterns, and laterality [11]; the psychology of human behaviour [12]; home care for women [13]; nursing care and health education [14]; recovery in speech disorders [15]; and the rehabilitation of patients who have experienced a stroke [16].

The concept of repatterning has been at the heart of the concept of ‘somatics’ since its inception in 1976 by Dr Tomas Hanna (1928–1990), the founder of Clinical Somatic Education [17,18]. It is an associative organism postulated by ISMETA, “The International Somatic Movement Education & Therapy Association (https://ismeta.org (accessed on 7 July 2023)), defined as a neuromuscular re-education strategy used in the artistic, therapeutic, and educational fields [17]. This makes the concept of repatterning a fundamental criterion of therapeutic practices of a somatic origin in relation to harmonious movement accompanied by sensory awareness and mindfulness [19].

The concept of ‘neuromuscular repatterning’ in dance was first described in the scientific literature by Krasnow in 1997 [20] as a neuropsychophysiological phenomenon resulting from the training of dancers based on a methodology termed ‘Imagery’. In this framework, neuromuscular repatterning is responsible for developing an improved motor response to technical gestures in dance, at the same time as promoting more creative mental states.

Dance is considered a physical activity in which the aesthetic function of behaviour is subordinated to high demands of effort. A lack of training program planning, poor technical execution, or the frequent performance of repetitive movements without sufficient recovery time [21] are factors that affect the psycho-physical behavioural balance of a dancer. This psycho-physical behavioural balance is altered as such an artist’s career progresses due to the lack of compensatory measures that require application [22]. Unfortunately, dancers with chronic or overuse injuries are more likely to ignore such injuries, sometimes resulting in more serious injuries and psychological distress [23]. The high injury rate associated with dance [24] is not a factor that precludes continuing to dance with inflammation, instability, pain, and discomfort [25]. A dancer’s necessity to survive economically and socially [24] and their need to avoid the disruption of the essential self through injury leads to the acceptance and development of pain tolerance [23]. In this regard, Dowd [22], highlights two essential psychosomatic factors that condition musculoskeletal disorders in dancers. Firstly, the optimal relationship between mental and neuromuscular activity, and, secondly, an adequate transfer of weight between the axes and their joint centres in relation to gravity, which is compromised by a change in psychophysical balance [26].

The subject of our study is neuromuscular repatterning, which is related to modulation between afferent and efferent pathways in humans [20]. This is a term explicitly used in the context of somatic movement education and therapy practices [17,27]. In these somatic methods, strategies such as external tactile or oral facilitation or the practice of employing symbolic mental representations are used [17]. In this context, the need to act on the prevention of injuries and the psychophysical well-being of dancers through intervention via somatic training programmes has already been described [28].

Therefore, our aim is to identify the key concept of neuromuscular repatterning in somatic training programmes for dancers.

## 2. Materials and Methods

### 2.1. Information Sources and Search Strategy

This systematic review was conducted according to the 2020 Preferred Reporting Items for Systematic Review and Meta-Analysis statement [29], collecting material from the inception of the databases until 13 October 2023 from Web of Science, PubMed, Scopus, Dialnet, SPORTDiscus, Sciences Direct, EbscoHost, and Google Scholar. For each of these databases, all possible combinations of the following search terms were used with the Boolean operator AND: Repatterning, Patterning, Somatic Practices, Dance, Health, Imagery, Developmental Movement, and Performing Arts. The final combinations of these terms were as follows: Repatterning AND somatic practices, Patterning AND somatic practices, Repatterning AND dance, Patterning AND dance, Repatterning AND health, Patterning AND health, Repatterning AND performing arts, Patterning AND performing arts. Imagery AND Dance, Imagery AND Performing Arts, Developmental Movement AND Dance, and Developmental Movement AND Performing Arts. The search was then extended by combining the terms Patterning, Patterned, Repatterning, and Patterner with Dance, Dancers, Ballet, or Dancing using the Boolean operator OR.

Well-being, Health, Injury Prevention, Overuse Injuries, or Somatic Training were used as complementary keywords in the databases to find an association with dance, as this topic could form part of another semantic or knowledge field in and of itself. These searches returned 1218 studies for review, some of which were duplicates or not relevant to the review. It was therefore necessary to extract the theme of our study, following a review tailored to the needs of the delimitation of the study objectives [30]. Systematic Review registration statement was created in PROSPERO (ID CRD42024502044, with the title ‘The Concept of Neuromuscular Repatterning in Dancers. A systematic review’).

### 2.2. Eligibility Criteria

The inclusion criteria utilized specified that the studies should (1) be primary sources (i.e., longitudinal and cross-sectional studies) or primary research sources, regardless of date of publication, given the specificity of the topic; (2) be published between the inception of the databases and October 2023; (3) be written in any language, with the material then being translated into English, or into any language with which the authors were fluent, when necessary (Japanese and Chinese studies were excluded on the basis of language proficiency); (4) be available as a full text; (5) be related to dance and somatic practices, in addition to improving the psychomotor functioning of individuals in the area of health application; and (6) address the concept of the concept of neuromuscular repatterning described explicitly or implicitly relating to mental and muscular activity to allow for the selection of an experiential methodology with dancers of any genre (Table 1).

### 2.3. Study Selection Process and Data Extraction

The selection process was divided into 4 distinct consecutive steps. First, all records retrieved from the databases were exported to Endnote Web, where duplicates were automatically removed. The next step was to merge all records into one database. Subsequently, titles and abstracts were assessed against the inclusion criteria by two co-authors. Finally, the full texts of the remaining records were screened by the same co-authors. At all stages, if there was disagreement between the two reviewers, a third reviewer made the final decision. The reference lists of all included studies were checked to ensure that no studies were omitted. Data from the included studies were collated by the first author in a specially created Microsoft Word document. The following characteristics were extracted: author and year of publication, keywords, type of sample, study design, training system, variables, type of evaluation, relevant results, and conclusions.

### 2.4. Study Risk-of-Bias Assessment

The Quality Assessment Tool for Quantitative Studies [31] was used by 2 researchers to assess the methodological quality of the included studies. The assessment tool consists of 6 components: (A) selection bias, (B) study design, (C) confounders, (D) blinding, (E) data collection methods, and (F) withdrawals. All components were rated on a scale of 1 to 3 according to different questions related to each section. The quality assessment tool dictionary was used to determine each score. Due to the heterogeneity of the included studies, component A was considered inappropriate for action-research and observational studies, and component D was considered inappropriate for experimental, action-research, and observational studies. Once all components had been assessed, the methodologies of the studies were classified as strong, moderate, or weak according to an overall rating. Studies were classified as strong if there were no weak component ratings, moderate if there was at least one weak rating, and weak if there were two or more weak ratings [31].

### 2.5. Synthesis Methods

To identify the studies’ designs, the classification proposed by O’Donoghue was implemented [32]. Studies were classified as observational; experimental (pretest and post-test randomized-group design, pre-experimental, ex post facto, pretest and post-test group design, or crossover); developmental (longitudinal prospective, longitudinal retrospective, or cross-sectional); survey; action-research; or case studies.

### 2.6. Certainty Assessment

The 2009 Oxford Center for Evidence-Based Medicine Levels of Evidence scale was used in order to determine the level of evidence of the included studies (https://www.cebm.ox.ac.uk/resources/levels-of-evidence/oxford-centre-for-evidence-based-medicine-levels-of-evidence-march-2009 (accessed on 14 October 2023)). Two co-authors graded the studies from Level 1b (the 2nd-highest level of evidence) to Level 5 (the lowest level of evidence). If there was disagreement, a third researcher made the final decision.

## 3. Results

### 3.1. Evolution of Results Screening

Following the application of the search term combinations, 836 results were returned, of which 382 were duplicates from the assigned databases, which were removed. For the next stages, the appropriate inclusion criteria were used to select the appropriate studies (Table 1).

For title screening, the search was restricted to original papers (inclusion criterion 1) and papers whose titles were associated with the study of dance and somatic practices (inclusion criterion 2). According to these criteria and based only on the first phase or selection by title, 56 studies were considered eligible.

During the abstract-screening stage, 42 studies were discarded as their topics were not contextualised with the field of improving psychomotor functioning (inclusion criterion 3). Therefore, there needed to be an allusion to both somatic and psychological aspects or to the scope of the associated terminology. During this phase, 14 studies were selected according to these criteria.

In the full-text assessment phase, it was necessary to verify that there was an effective methodology for dancers of any genre, reflecting practical content or training programmes in which the concept of neuromuscular repatterning was described in relation to movement variables (inclusion criterion 4). In the remaining studies, it was possible to infer or observe the results of these somatic methods (inclusion criterion 5).

Following the application of the inclusion criteria, five studies were deemed eligible for this systematic review. All of them pursued our aim, i.e., to establish a relationship between the concept of neuromuscular repatterning and health aspects related to functional technical improvement, psychosomatics, and positive impacts on the management of dance injuries.

**Figure 1:**
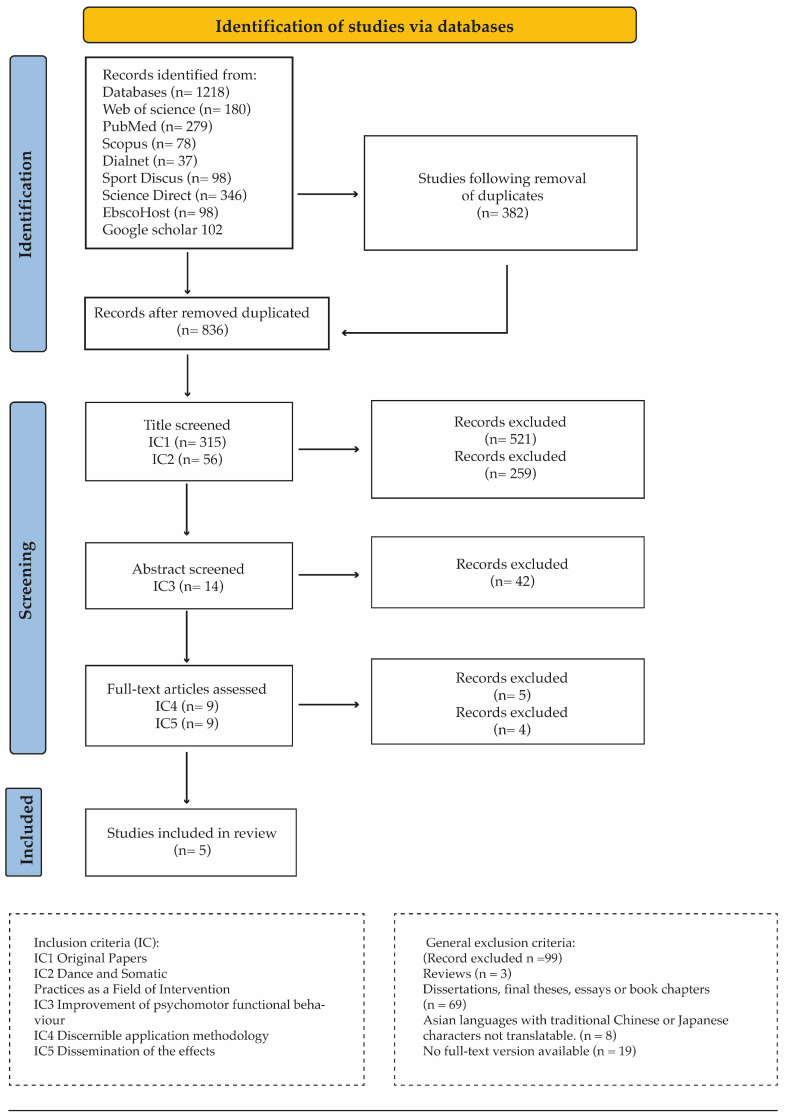
PRISMA (Preferred Reporting Items for Systematic Review and Meta-Analysis) flow diagram showing the selection process followed [29].

The PRISMA diagram outlines the process followed to focus the fields of study and apply the inclusion and exclusion criteria to determine the relevant scientific publications that determine the background of the neuromuscular substrate of the repatterning concept and the effect of psychosomatic functional health on dancers (Figure 1).

### 3.2. Study Selection

Table 2 reveals that a total of 1218 studies were retrieved; following the removal of duplicates, 1038 abstracts remained, of which 14 were eligible for full-text assessment after title and abstract screening. During the full-text assessment, 723 records were excluded as they did not meet the eligibility criteria. Finally, after full-text screening, five studies related to the concept of neuromuscular repatterning in the field of somatic training programmes and its relationship with practical application to dancers were included in the systematic review [33,34,35,36,37].

### 3.3. Study Characteristics

The included studies were published between 1997 and 2010 (Table 3, Table 4 and Table 5). The samples were students engaged in junior (four studies) and senior (one study) university dance programmes. All the participants were engaged in university dance programmes in the USA (four studies) and UK (one study). However, 40% of the studies did not describe the number of participants who had undergone a somatic training programme (Table 4). In addition, most of the participants had a background in different dance genres (four studies) that allowed them to take university courses or pursue university degrees not specialised in genres such as classical ballet, although this technique was taught in their university dance programmes.

### 3.4. Studies’ Authors and Sources of Publication

Table 3 reports the five studies that demonstrate practical experiences of somatic training in dance [33,34,35,36,37].

Table 4 describes the objectives and methodologies pursued in each study. The studies reflect the types of influences the researchers wanted to provoke in their samples of dance practitioners. Among the most relevant ones, common to somatic practices, we highlight the following: (1) body alignment [36]; (2) awareness of body processes such as breathing, sensing, and connection to and initiation of the practice of developmental movement [33]; (3) neurodevelopmental foundations [34]; (4) dance technique acquisition processes between teacher and student [37]; and (5) psychosomatic mindfulness of dancers [35].

Table 5 presents the variables directly related to health, highlighting (1) integration and inter-articular connectivity in movement [36,37]; (2) the neurological component of alignment and efficiency during dance practice [33,35]; (3) anatomical experience as a basis for communication and social well-being in a class [34]; and (4) self-confidence [35,37].

Table 6 reports on the studies’ quality in relation to the Oxford Levels of Evidence. One study reported a strong global rating of quality, while three studies reported a weak global rating, and one study reported a moderate global rating. Four studies were not checked for potential confounders, and only one study used valid and reliable data collection methods. Furthermore, selection bias criteria were applied to only one study, and the scores were moderate in one study and weak in the three remaining studies. Withdrawals were described in one study, and blinding criteria were deemed inapplicable for all studies. Overall, one study was rated as Level 1b, while two studies were rated Level 2c and another two Level 5 on the Oxford Levels of Evidence scale.

## 4. Discussion

Our aim was to identify insights into the key concept of neuromuscular repatterning in somatic training programmes for dancers. Weber reviewed the academic literature on the incorporation of somatic practices in educational and dance programmes and provided a profound reflection on the process of incorporating somatic concepts into dance [37]. Generally, we can distinguish two major methodologies underlying the ‘key concept’ of neuromuscular repatterning in somatic training programmes for dancers, namely, developmental movement [18,38,39] and imagery [22,38,39,40,41].

### 4.1. Developmental Movement

Krasnow et al. explain how deviations from central vertical alignment are highly variable depending on the prevailing movement conditions, but they do not explore whether alignment dynamics can influence dancers’ motor performance in dance [36]. However, it is clear that the recovery of an efficient vertical alignment axis is related to a dancer’s level of proprioceptive competence [42]. Dancers are neurologically reprogrammed as they progress through these milestones of pursuing verticality and gait [39]. A dancer, like an infant, passes over floor surfaces, which provide a great deal of support and contact, proprioceptively facilitating a new neurological organisation when reaching verticality [42].

This methodology, based on the principles of developmental movement, suggests a rethinking of the rebirth of an initial upright position. From a developmental perspective, an infant strives to stand upright in the early stages of life and can reach a standing position by approximately one year of age, and this process facilitates the search for effective balance [22]. As early as 1993, Cohen described an infant’s functional developmental model as part of a methodology called developmental movement [39].

This method consists mainly of re-experiencing or modifying neurodevelopmental patterns typical of childhood during adulthood. This methodology has been applied in actor-training programmes through a psychosomatic approach [18] with transfers to performance [27]. It has also been formally used for the acquisition of motor skills in educational programmes in physical education [43].

The aim of this evolutionary rethinking is to reorganise a new, more efficient, and healthy verticality, which is a common goal regardless of the somatic technique used [44]. Developmental movement can also be a reference model for any dance practitioner.

Brodie and Lobel (2004) [33] use the fundamentals of developmental movement pertaining to the Alexander Technique, the Feldenkrais Method, Laban/Bartenieff Movement Analysis, and Body–Mind Centering. In this sense, Brodie and Lobel [33] emphasise the importance of somatic training for improving alignment. It is the neurological component of alignment that enables the effectiveness of the technical response during dance practice [33,35,36] (Table 5).

The central intention in Evolutionary Movement practice is to explore the maximum range of expressivity through sensation rather than effort and to progressively increase that range [18].

In her sessions, Eddy implements a structure in which neurological reorganisation is utilised with the neurodevelopmental concepts belonging to the neurodevelopmental fundamentals of Body–Mind Dancing: the union of Body–Mind Centering and Laban Movement Analysis/Bartenieff’s Fundamentals [34] (Table 4). These sessions incorporate floor- and spatial-level work and allude to the history of movement from infancy and its connection to neurodevelopmental movement skills. Eddy [34] associates it with accurate and higher-quality communication.

The key concepts of neurodevelopment are applied in Weber’s 2009 work [37] in relation to work on Body–Mind Centering with regard to the contemporary dance technique. Weber demonstrates a clear development of body connection, increased creativity, and self-confidence in the dancers’ movements. Kearns 2010 [35] expresses these neurodevelopmental concepts through Bartenieff’s fundamentals. Kearns [35] determined that alignment is a variable associated with a mind–body connection, with mindfulness transferring to the rest of the class and allowing the dancer to incorporate it into rehearsal and performance.

However, regarding the concept of organicity and repatterning, Brodie and Lobel express that most dancers have experienced a strange sensation when they have reorganised themselves correctly in the vertical direction [33]. It is known that there is a temporary period of qualitative adaptation when an individual reorganises neurologically. In this regard, Krasnow et al. developed a method for assessing central vertical alignment while an individual is immobile [36] (Table 5). However, they do not apply neurological reorganisation fundamentals based on developmental movement.

In our results included in this review, four out of five studies [33,34,35,37] used methods that incorporate strategies, elements, or fundamentals belonging to the developmental movement system as part of the aim of neuromuscular repatterning, which is neurological reorganisation in the field of the application of somatic practices applied to dance.

### 4.2. Imagery

Imagery is a type of strategy that can take the form of guided imagery, reflex movements initiating voluntary movements, or the visualisations themselves accompanying a simulation of technical sporting [45,46,47], dance [48,49,50], or aesthetic sporting [46] gestures to improve performance [51].

Imagery is known as a facilitating resource for a dancer’s creative intervention in choreographic processes [52] and movement improvisation [53]. Pavlik and Nordin-Bates allude to the figure of Lulu Sweigard (1895–1974) as the creator of the somatic facilitation technique termed Ideokinesis [54]. Ideokinesis is described as the best-known method of neurological rehabilitation in dance and as a mental intervention used to facilitate movement for injured dancers [55].

All of the selected somatic training programmes in our results [33,34,35,36,37] use the Imagery or Imagery Guided System, with a basic substrate of the anatomy and physiology of the body systems.

Krasnow et al. [36] constructed an imagery System for dancers based on the joint synthesis of the work of Irmgard Bartenieff [38], Irene Dowd [22], and Lulu Sweigard [40] (Table 4). Krasnow et al. emphasise that imagery is the appropriate working direction to transfer the improvement of alignment from a situation of rest, a fixed posture, to situations of movement dynamics in dance (Table 5). Krasnow [20] suggested that one of the reasons dancers seek alternative training methods is the high rate of injury in the dance population. Many dancers seek methods of intervention and rehabilitation outside of the medical field.

Nordin-Bates et al. link psychological factors to injuries among dancers, highlighting the prevalence of musculoskeletal injuries, especially among professionals, suggesting the need for improved treatment and training with psychological techniques. They recommend integrating imagery into dance curricula [56]. This was suggested by Krasnow, who highlighted the need for the ongoing psychological assessment of the dance population due in part to the stress they experience [20].

In their somatic training programme, Brodie and Lobel 2004 [33] use an imagery system based on ldeokinesis wherein the process of teaching dance from experiential anatomy is drawn on metaphorically [Table 4]. This forms a fundamental part of the didactic approach to integrating these somatic practices into dance technique classes. The application of this imagery System is perceived to be more beneficial than a directive style correction in terms of improving responsiveness during movement as well as alignment and competence in dance performance skills. [Table 5].

Eddy (2006) [34], in her somatic training programme, applies visualisation to dance through the training of body systems such as the skeletal, muscular, and nervous systems [Table 4]. Eddy [34] observes that this work of anatomy and experiential physiology benefits a dancer’s communicative and expressive potential [Table 5].

Weber (2009) [37] also uses experiential anatomy and physiology work, but with a focus on body fluid systems in dancers [Table 4]. Weber observes [37] in his semi-structured somatic practice more empowerment and enjoyment than in more dance-directed methodologies, thus generating an enhanced sense of well-being [Table 5].

Finally, Kearns 2010 [35] used the work of ‘Ideokinesis Facilitation’ as a strategy for mental concentration on visualised anatomical actions. Kearns [35] observed improvements not only in variables such as body alignment but also in the fluidity and mindfulness of movement during dance. Kearns [35] used a methodological implementation called Somatic in the floor barre, a work of integrative somatic techniques (such as Ideokinesis and Pilates), which has the effect of muscular repatterning as one of the most important achievements in its application of pelvic exercises in the written self-evaluation of students. These reports demonstrate that short periods of rest on the floor are also essential to allow the body and mind to absorb deep neuromuscular work and for the more subtle repatterning of movement to occur. Batson describes how somatic practice can illuminate how to focus on the function of the trunk as the basis for supporting and articulating the limbs in a technical element of battement tendu [55].

In all of the selected studies [33,34,35,36,37], the purpose of Ideokinesis is based on the idea that imagery can improve skeletal alignment and fluidity of movement by repatterning neuromuscular pathways in the absence of overt movement. In addition, the authors each have their own reference sources on which to base the rationale for their somatic training programmes [33,34,35,36,37].

Krasnow et al. [36] include several types, such as anatomical and metaphorical imagery; lines of movement; and global, visual, or kinaesthetic imagery. Brodie and Lobel [33] and Eddy [34] use informative feedback on anatomy, kinesiology, and physiology at the beginning and end of each programme session. Weber [37] based their feedback on the physiological systems of the body, namely, the cerebrospinal, synovial, lymphatic, and circulatory systems, based on the work of Eddy [34]. Kearns (2010) [35] focuses on the analysis of anatomical systems.

The methodologies of all the selected programmes [33,34,35,36,37] use human anatomy and physiology to maximise dancers’ expression of movement.

In this process, neuromuscular repatterning is employed to develop psychophysical capacity and potential from the collection of sensations to improve the motor responses pertaining to the technical gestures in dance and promote more creative mental states [34]. Imagery is used in somatic training programmes [33,34,35,36,37] by embodying our own organic systems. This work seems to have an effect on the modulation and reorganisation of dancers’ bodies as they explore through movement.

According to our results (Table 3, Table 4 and Table 5), the framework of somatic training programmes for dancers can be semi-structured or integrated into the acquisition of the dance technique itself [33,35,37]. This type of methodology is more targeted than open self-discovery frameworks [34], and the application framework can be extended to the actor’s performance on stage [27].

All the included studies [33,34,35,36,37] apply a process of neurological reorganisation underlying repatterning that needs to be identified as a fundamental concept in somatic practices. Krasnow et al. [36] address it in imagery work content to better assimilate physical conditioning content. Brodie and Lobel [33], Eddy [34], and Weber [37] apply this methodology during the acquisition of dance techniques in a more transversal way throughout all sessions, while Kearns [35] focuses more on its application in the initial part of a session [35] (Table 4).

In all somatic approaches, a student’s autonomy guides somatic awareness, and seeking wellness, preventing injury, and optimising neuromuscular repatterning are considered. Krasnow expresses the regularity of finding dancers participating in somatic and conditioning programmes outside of traditional dance classes that improve dance performance and reduce the risk of injury [20].

Eddy suggested that most somatic dance training protocols are aimed at a broader population or domains of community dance, integration, and holistic or socio-psychological awareness of the field of action in which they are applied [28]. In our systematic review, the five experiences found are situated in a mainly educational and academic context [33,34,35,36,37].

In this context, we find the application of two major training systems: developmental movement and imagery. In addition, the Experiential Anatomy and Physiology of Body Systems combines the understanding not only of imagery in the reorganisation of new motor movement programmes but also of developmental movement as an inherent part of human beings.

Neuromuscular repatterning is a process that achieves a modulation of new motor patterns by allowing a greater pre-activation of sensory pathways and a new awareness of the activation or inactivation of efferent motor pathways.

### 4.3. Study Limitations

The studies included in this review combine quantitative and qualitative research, which makes the application of the Quality Assessment Tool for Quantitative Studies and the Oxford Levels of Evidence Scales difficult. Despite this, and due to the small number of studies that met the criteria, the authors applied these two assessment tools.

## 5. Conclusions

The dominant concepts underlying the results of this research are mental training and sensory awareness training. In the training of dancers, this sensory training must be preceded by motor training that induces neuromuscular repatterning.

Neuromuscular repatterning may compensate for or restore habitual or poorly acquired aesthetic behavioural patterns due to the high work demands associated with dance.

Developmental movement and imagery are two methodologies strongly connected to the concept of ‘neuromuscular repatterning’ in somatic training programmes for dancers. The performance of further quantitative experimental or quasi-experimental studies is warranted to better define the level of improvement or impact of neuromuscular repatterning with respect to dancers.

## Figures and Tables

**Table 1:** Stages of review and associated criteria.

Stages	Process
Database search	Search of Web of Science, PubMed, Scopus, Dialnet, SPORTDiscus, Sciences Direct, EbscoHost, and Google Scholar
Title review	Confirmation that the title of an article was relevant to dance and somatic practices to allow for the inclusion of original papers
Abstract review	Using the databases to select only studies in the area of improving psychomotor functioning with discussion of both somatic and psychological aspects of health application field
Full text review	Assessing the concept of repatterning (neuromuscular), described explicitly or implicitly, relating to mental and muscular activity and associated experiential methodologies concerning dancers of any genre

**Table 2:** The studies selected and their sources.

Database	Framework	Title Review	Abstract Review	Full Manuscript Review
Web of Science	180	1	1	0
PubMed	279	1	0	0
Scopus	78	5	2	2
Dialnet	37	3	1	0
SPORTDiscus	98	9	3	2
Science Direct	346	3	1	0
EbscoHost	98	10	6	1
Google Scholar	102	24	0	0
TOTAL	1218	56	14	5

**Table 3:** Studies’ authors and sources of publication.

Study	Authors (Year)	Title	DOI	Journal	Keywords
1	Donna H. Krasnow, Steven J. Chatfield, Sherrie Barr, Jody L. Jensen and Janet S. Dufek (1997) [36]	Imagery and Conditioning Practices for Dancers	10.2307/1478236	*Dance Research Journal*, 29(1):43–64	Training systems, dance technique, dance skills, neuromuscular repatterning, muscular/structural alterations
2	Julie Brodie & Elin Lobel (2004) [33]	Integrating Fundamentals Principles Underlying Somatic Practices into the Dance Technique Class	10.1080/15290824.2004.10387263	*Journal of Dance Education*	Exteroceptive and proprioceptive systems, movement problems, Alexandre Technique, Feldenkrais Method, Laban/Bartenieff Movement Analysis, Body–Mind Centering, Ideokinesis
3	Martha Eddy (2006) [34]	The Practical Application of Body-Mind CENTERING (BMC) in Dance Pedagogy	10.1080/15290824.2006.10387320	*Journal of Dance Education*	Laban Movement Analysis/Bartenieff Fundamentals (LMA/BF), embodied practices, experiential anatomy, neurodevelopmental foundation movement re-education
4	Rebeca Weber (2009) [37]	Integrating semi-structured somatic practices and contemporary dance technique training	10.1386/jdsp.1.2.237_1	*Journal of Dance of Somatic Practices*	Somatic movement, dance education, teaching practices
5	Lauren W. Kearns (2010) [35]	Somatics in Action. How ‘I feel Three-Dimensional and Real’ Improves Dance Education and Training	10.1080/15290824.2010.10387158	*Journal of Dance Education*	Rehearsal process, educational environment, alignment, Yoga, Pilates, Bartenieff Fundamentals, Ideokinesis mindful movers

**Table 4:** Objectives and methodologies.

	Authors (Year)	Objectives	Sample	Study Design	Training System
1	Krasnow et al. (1997) [36]	- To develop and test measurement tools that could be used to evaluate the effects of conditioning with imaging on dance performance and body alignment - To evaluate the influence of conditioning with imaging on dance performance - To evaluate the influence of conditioning with imaging on body alignment for dance - To incorporate anatomical and metaphorical images, lines of movement, and global, visual, and kinesthetic images (Imagery System)	Twenty university dance students (n = 20)	- Observational analysis study conducted by previously trained experts through a pilot study - Experimental (pretest post-test group design)	- Imagery System: Synthesis of the work of Irmgard Bartenieff, Irene Dowd, and Lulu Sweigard. - Conditioning system: Synthesis of the work of Fitt, Pilates (from Friedman and Eisen), Rommet, and Solomon - Conditioning-with-imaging system: a system combining both, involving the subjects in movement and imagining simultaneously
2	Brodie J & Lobel E (2004) [33]	- To introduce the fundamental principles underlying somatic practices - To provide a didactic model based on 4 principles to integrate the corresponding ideas into dance technique lessons	Undergraduate students in the Dance, Drama, and Film Department at Kenyon College, Gambier, Ohio USA	- Observational	The 4 principles based on awareness of breathing, sensing, connecting, and initiation regarding developmental movement are part of the training, namely, the Alexander Technique, Feldenkrais Method, Laban/Bartenieff Movement Analysis, Body–Mind Centering, and Ideokinesis, wherein the process of dancing from experiential anatomy is drawn on metaphorically.
3	Eddy M (2006) [34]	- To apply theoretical components belonging to the neurodevelopmental fundamentals of BodyMind Dancing © to dancers.	Undergraduate students from Empire State College New York (NY) City, New York State, or belonging to NY dance academies.	- Observational	Training of body systems such as skeletal, muscular, and nervous systems applied in Body–Mind Centering through improvisation and dance visualisation, with frequent applications to movement and dance.Body–Mind Dancing, union of BMC with LMA/BF applied in 6 phases (see Keywords, Table 3)
4	Weber R (2009) [37]	- To analyse how a semi-structured somatic practice in a contemporary dance technique lesson at different teaching levels affects dance students. - To investigate how to incorporate the analysed somatic practice into a contemporary dance technique lesson. - To improve the author’s personal teaching practice and transmit this information to interested educators, offering what in the academic world of dance education has been called ‘local and public knowledge’	7 students (n = 5 females/n = 2 males aged 16 to 37 years old). Background in different genres This sample were in Year 1 at the Performing Arts (Dance) National Diploma Program at Warrington Collegiate (England, United Kingdom)	- Action research	Training is based on the following: Body–Mind Centering focus fluid systems. - Contemporary Technique
5	Kearns LW (2010) [35]	- To analyse how somatic movement practices (integrated by multiple somatic theories, such as yoga-based alignment, Pilates, Bartenieff’s Fundamentals, and Ideokinesis) impact dancers	n = 12 Junior- and senior-level students from the Dance program at Elon University, Elon, North Carolina	- Action research	Four disciplines for sustaining mindfulness in dancers in a floor barre: - Pilates method for postural alignment and balance. - Yoga based on physical alignment - Bartenieff fundamentals. - Ideokinesis—mental concentration on imagined anatomical actions.

**Table 5:** Variables, scales of measurement, and results.

	Authors (Year)	Variables	Evaluation: Instruments, Scales, and Measures (*)	Relevant Results	Conclusions
1	Krasnow et al. (1997) [36]	I. Dance performance (4 categories): a. Full body involvement in the movement—axial, locomotor, and limb energy b. Body integration and connectedness in movement c. Articulation of joints and body segments—lower-limb and upper-limb activity d. Movement skills in dance—direction and level changes, balance, speed, and dynamic and movement quality II. Body alignment: vertical and central alignment	- Movement Imagery Questionnaire developed by Hall and Pongrac in 1983 - Questionnaire for medical screening - Pre-test from Dance Performance Measure (PCEM). - Pre-and-Post Tested: I. The Performance Competence Evaluation Measure (PCEM) modification of the Aesthetic Competence Evaluation (ACE) (Chatfield and Byrnes, 1990). II. The Dynamic Alignment Measure (DAM) was developed according to the performance of a grand plié	(I.) Dance Performance The overall scores (4 categories: a, b, c, and d) were improved globally, with Group 1—Imagery showing the greatest improvement. Time had a significant impact (*p* = 0.0003) In the results of II: the body alignment results suggest that the findings form the study of alignment in a resting or stationery position are not transferable to real conditions in dance practices. This demonstrates that under real conditions, there is continuous adaptation to changes in position and balance. Alignment from a previous movement sequence based on modern dance codes involves greater variability and dispersion in relation to the reference axis	I. Dance Performance The lack of significant results between groups reveals the need for a psychological evaluation, such as a standardized stress test, (pre- and post-testing), as many dancers are undergoing very stressful academic periods. These emotional states fluctuate in dancers, and it is difficult to determine the effects of these emotional states on the results. II. Body Alignment The combined imagery and conditioning training group revealed better results in improving alignment The imagery training group revealed that it takes longer than 8 weeks for neurological changes to evolve into complex movements. The results suggest that training sessions should be extended over longer periods of time, particularly when addressing the neural components of alignment and movement
2	Brodie J & Lobel E (2004) [33]	Sensitivity, awareness, and responsiveness during movement Alignment, efficiency, and competence for a dance and performance skills class	Observational process and direct first-person experience with dancers engaged in dance academy training	Verbal feedback during the dance technique class, given through directed questions, provides imagery or suggests desired kinesthetic sensations that are more beneficial than a direct command genre correction	This methodology facilitates the transfer of these somatic concepts The ultimate goal of focusing on breath, connection, sensation, and initiation while dancing is to increase awareness, an awareness that opens the door to making new choices. Dancers are informed by their kinaesthetic sense and the external environment Being aware of movement leads to a reflective and satisfying interpretation in relation to a psychosomatic state of personal well-being
3	Eddy M (2006) [34]	The dancer’s expressive and communicative potential	Observational and first-person direct experience process with dancers engaged in dance academy training	Dancers benefit from the precision and embodied practice of their experiential anatomy, allowing them to communicate with greater quality and precision in genres taught by a choreographer or teacher	Experiential physiology and anatomy are pathways to diversity of expression and allow students to express various aspects of themselves. This reflects a state of personal well-being and improved psychosocial relationships in dance classes
4	Weber R (2009) [37]	Creativity Critical understanding Confidence Development of technique–body connection	Subjective observations of the researcher Multiple data sources and collection methods, which included video and audio recording of sessions, transcriptions of group discussion (open interview) feedback, and participant observations made by both the author and the usual course tutor	The students demonstrated a clear development of body connection, greater creativity in their body expression with respect to their movements, increased levels of confidence, and a critical understanding of the basic principles of movement awareness, with greater involvement of technique	Within the context of the application of this semi-structured somatic practice between somatic practice and dance technique training, the students become more aware of embodying their own bodies. This translates into feeling more empowered and experiencing greater enjoyment, with a greater sense of well-being Any harmful elements in the approach to technical patterns are avoided in favour of a healthy practice
5	Kearns LW (2010) [35]	Alignment Self-confidence Cohesive, fluid, and mindful movement	Self-assessment reports made by students Subjective observations made by the researcher	Using a significant portion of class time on the floor barre allowed students to reinforce their mind–body connection as well as the transfer of mindfulness to the rest of the class. Improvements were seen in the students’ approach to movement, mindfulness, and expressiveness both in the classroom context and in rehearsals and performances.	Based on the researcher’s observations and self-analyses of their experience, the analysed method, Somatic in Actions, helped the dancers to reflect on and articulate their abilities to achieve both subtle and complex movements. in their performance and in the practice of self-awareness that accompanies the development of their processes as a dancer of the 21st century

(*) The evaluative tests incorporate the dates of their implementation (as described by the author) found within our selection of results.

**Table 6:** Results of the quality assessment scores (global ratings) and Oxford LoE from the included studies.

Study	Quality Assessment Scores (Global Rating)							Oxford LoE from the Included Studies
	Selection Bias (A)	Study Design (B)	Cofounders (C)	Blinding (D)	Data (E)	Withdrawal (F)	Global Rating	LoE
Krasnow et al. (1997) [36]	2	4	2	2	1	1	1	1b
Brodie & Lobel (2004) [33]	1	4	4	3	3	1	3	5
Eddy (2006) [34]	1	4	4	3	3	1	3	5
Weber (2009) [37]	1	4	4	3	2	1	2	2c
Kearns (2010) [35]	1	4	4	3	3	1	3	2c

Note: ‘Data’ refers to data-collecting methods. The quality assessment scores criteria were as follows: 1, strong; 2, moderate; 3, weak; and 4, not applicable. The LoE was classified from 1, the highest level, to 5, the lowest level. Please see the ‘Section 2’ for more details about the applied criteria and the assessment of both global ratings (via The Quality Assessment Tool for Quantitative Studies) and LoE (via the Levels of Evidence scale).

## Data Availability

The data presented in this study are available on request from the corresponding author.
